# Solution of Bi-chloride of Mercury in Itching

**Published:** 1841-02

**Authors:** 


*Solution of Bi-chloride of Mercury in Itching.*—Dr. Prus states that he has often succeeded in the treatment of prurigo, an affection often obstinate, in the old men under his care in Bicêtre, by means of lotions night and morning, over all the parts where the pruriginous papules exist, with a solution of bichloride of mercury, one drachm in half a pint of water.

Mr. Duparque employs with benefit the same solution in pruritus of the internal surface of the labia pudendi, which occurs in some females in the decline of life, and which is often intolerable and difficult of removal.—*Jour. de Connaiss. Méd.*, May, 1840.—*Ibid.*

